# Configurational Entropy Approach to the Kinetics of Glasses

**DOI:** 10.6028/jres.102.011

**Published:** 1997

**Authors:** Edmund A. Di Marzio, Arthur J. M. Yang

**Affiliations:** National Institute of Standards and Technology, Gaithersburg, MD 20899-0001; Armstrong World Industries, 2500 Columbia Ave., Lancaster, PA 17603

**Keywords:** Gibbs-Di Marzio entropy theory, glass kinetics, glass transition temperature, Vogel equation

## Abstract

A kinetic theory of glasses is developed using equilibrium theory as a foundation. After establishing basic criteria for glass formation and the capability of the equilibrium entropy theory to describe the equilibrium aspects of glass formation, a minimal model for the glass kinetics is proposed. Our kinetic model is based on a trapping description of particle motion in which escapes from deep wells provide the rate-determining steps for motion. The formula derived for the zero frequency viscosity *η* (0,*T*) is log *η* (0,*T*) = *B* − *AF*(*T*)*kT* where *F* is the free energy and *T* the temperature. Contrast this to the Vogel-Fulcher law log *η* (0,*T*) = *B* + *A*/(*T* − *T*_c_). A notable feature of our description is that even though the location of the equilibrium second-order transition in temperature-pressure space is given by the break in the entropy or volume curves the viscosity and its derivative are continuous through the transition. The new expression for *η* (0,*T*) has no singularity at a critical temperature *T*_c_ as in the Vogel-Fulcher law and the behavior reduces to the Arrhenius form in the glass region. Our formula for *η* (0,*T*) is discussed in the context of the concepts of strong and fragile glasses, and the experimentally observed connection of specific heat to relaxation response in a homologous series of polydimethylsiloxane is explained. The frequency and temperature dependencies of the complex viscosity *η* (*ω*< *T*), the diffusion coefficient *D*(*ω*< *T*), and the dielectric response *ε* (*ω*< *T*) are also obtained for our kinetic model and found to be consistent with stretched exponential behavior.

## 1. Introduction

In this paper we first critically review the entropy theory of glasses. After defining a glass in Sec. 1.1 we show in Sec. 1.2 the need for an equilibrium thermodynamic theory of those materials that form glasses. Sec. 1.3 gives our reasons for believing that the vanishing of the configurational entropy *S*_c_, or at least the entropy reaching a critically small value, is associated with glass formation. Sec. 1.4 describes briefly the many experiments that support the entropy theory of glass formation. Sec. 1.5 offers a critique of equilibrium theories. In Sec. 1.6 the suggestion is made that the *S*_c_ = 0 criterion can be replaced by *S*_c_ = *S*_co_. *S*_co_ is a small critical value of the entropy which is dependent on the time scale of the experiment but is positive even for infinitely long time scale. Sec. 1.7 contains qualitative insights into the kinetics of glass formation arising from the *S*_c_ → 0 criterion, while Sec. 1.8 makes the observation that the fluctuation-dissipation theorem provides quantitative insights into the connection between the equilibrium and kinetic properties of glasses.

The kinetic theory is developed in Sec. 2. In Sec. 2.1 we pass from phase space to configuration space and gain an insight into the topology of configuration space. In Sec. 2.2 we use the principle of detailed balance to evaluate the transition rate constants of the master equation describing minimal models of glass formation. In Sec. 2.3 using a trapping model for the phase point we define these minimal models and derive their associated (master) equations. Sec. 2.4 contains derivations of the zero frequency diffusion coefficient *D*(0,*T*) and complex viscosity *η* (0,*T*), while in Sec. 2.5 frequency dependent *D*(*ω*< *T*), *η* (*ω*< *T*) and the dielectric response *ε* (*ω*< *T*) are obtained. These quantities each depend on the distribution of well depths *W*(*E*). This quantity *W*(*E*) = exp (*S*_c_/*k*) is discussed in Sec. 2.6 where viscosity is shown to be a function of free energy. Finally, Sec. 3.1 discusses our results while Section 3.2 offers some conclusions.

### 1.1 Operational Definition of a Glass

We define a glass to be a material which is an ordinary liquid at high temperatures and whose thermodynamic extensive quantities, volume *V*, and entropy *S*, fall out of equilibrium as we lower the temperature past some temperature *T*_g_ which depends on the rate of cooling. Above *T*_g_ the relaxation times associated with viscosity are less than the time scale of the experiment, while below *T*_g_ they are greater. The above definition describes the formation of a crystal as well as a glass so we augment our definition by requiring that the extensive thermodynamic quantities be continuous at *T*_g_ and that there be no change of spatial symmetry as we cross *T*_g_. This operational definition immediately suggests a number of questions which must be answered if we are to understand glasses. 1) What are the *V*(*T*,*P*) and *S*(*T*,*P*) equations of state on the high temperature side of *T*_g_? 2) For a given rate of cooling, why does the glass transition occur at one temperature, *T*_g_, rather than some other temperature? 3) What are the thermodynamic properties well below *T*_g_ where the relaxation times for diffusion of molecules are so long that some degrees of freedom are frozen out and only oscillatory motions occur? Experimentally the glass is known to behave like an elastic solid. 4) What is the viscosity *η* (*ω*< *T*,*P*), where *ω* is frequency? The first three questions are concerned exclusively with the equilibrium properties of glasses.

### 1.2 Necessity for an Equilibrium Theory of Those Materials That Form Glasses

There are four bona fide reasons to formulate an equilibrium theory of glasses [1, 2]. They are:

1. Glasses have equilibrium properties above *T*_g_ and well below *T*_g_. It is sensible to ask what they are.
2. The crystal phase is not ubiquitous. This proposition was proved in Ref. [2]. Therefore, an equilibrium theory is needed for the low temperature phase which we know is not a crystalline phase. Of course, thermodynamics is also needed to describe the low temperature metastable phase of those materials that can crystallize.
3. An equilibrium theory is needed [3–5] to resolve Kauzmann’s paradox [6, 7]: An equilibrium theory allows us to extrapolate equilibrium quantities through the glass transition to see how the “negative entropy” and “volume less than crystal volume” catastrophes are avoided even when the experimental relaxation times are projected to be infinite. For polymer glasses the sharp leveling off of the experimental thermodynamic quantities must also occur in a correct equilibrium theory. This either is a second-order transition or it approximates one. Either case allows us to calculate a *T*_2_ to which the *T*_g_ tends in very long time-scale experiments.
4. An equilibrium theory is a necessary prerequisite for an understanding of the kinetics [7].

### 1.3 Vanishing of Configurational Entropy is the Thermodynamic Criterion of Glass Formation

Once one is convinced that the equilibrium properties of glassy materials exist there are no options. One simply evaluates the partition function and then the two equations of state *V*(*T, P*) and *S*(*T, P*). It is required, of course, that the important characteristics of the molecules be taken into account, at least within a minimal model. This minimal model (the simplest model which retains the essence of the problem) must have both intermolecular energy to allow for volume changes and intramolecular energy to allow for temperature dependent shape changes of the molecules. The lattice model of Gibbs and Di Marzio [3–5] (GD) is a minimal model for polymers which incorporates an intermolecular bond energy *E*_h_ which regulates the number of empty lattice sites (volume) and an intramolecular stiffness energy Δ*ε*< which controls the temperature dependent shape changes. When this was done within the framework of the Flory-Huggins (F-H) approximation it was discovered that a second-order transition in the Ehren-fest sense was obtained and that the *T*(*P*) line separating the liquid state and the glassy state was given by the vanishing of the configurational entropy

$$S_{c}\left(T_{2},P_{2}\right)=0.$$ (1)

The basic physics behind glass formation in polymers is as follows. At high temperatures, because of the (semi-) flexibility of the polymers and the large numbers of holes, there are many ways to pack the molecules together in space. At these temperatures the interferences among the molecules are not of the kind that prevent the molecules from taking up their preferred shapes; if the internal energy associated with shape *i* is *E_i_* then the probability of observing shape *i* is proportional to exp(−*βE_i_*). As we lower the temperature the configurational entropy approaches zero. The individual molecules now can no longer continue to achieve their Boltzman shapes (the shapes implied by the Boltzman distribution of internal energies) for as the mathematics show this would imply that *S*_c_ ≤ 0, which is an impossibility. Instead the molecules are frustrated [8] by their neighbors from achieving their individual Boltzmann shape distributions and at lower temperatures (*T* ≤ *T*_2_) characteristic of the glassy region the distribution of shapes of the molecules is given by the Boltzmann distribution at *T*_2_.

#### Liquid Crystal Frustration

This interpretation is strengthened by our understanding of the isotropic to nematic phase transition occurring in a system of rigid-rod molecules. At low concentrations of an isotropic distribution of rigid-rods the entropy is large because the rigid-rods have both orientational and translational freedom. However, as the rigid-rod concentration increases these freedoms begin to disappear until at a critical concentration there is no longer any freedom for the rigid-rods to rotate or translate provided only that the distribution of orientations is random. This is the point where the configurational entropy approaches zero (there may be small pockets where a trapped rigid-rod can partially rotate or translate slightly). One can gain much insight into this problem by packing pencils or soda-straws at random on a table-top (this is the two dimensional problem) or piling together rigid sticks obtained from pruning one’s garden (this is the three-dimensional problem). It immediately becomes obvious that there is a critical density above which one can not go if the rods are to remain isotropically distributed in space. This critical density is given approximately by *v_x_* = *C*/*x*, where *x* is the asymmetry ratio of the rods and *v_x_* is the volume fraction of rods. The constant *C* is about 4 for one lattice model [9] and 8 for another [10]. For straight rigid-rods the system has a way out of the packing difficulty; the rods can align and do so forming the nematic phase [11]. *The ordered phase has a larger entropy than the disordered phase* because as the reader can readily verify by a simple table top experiment (partially) ordered rods gain both translational and rotational freedom!

#### Packing of Semi-Flexible Polymers

Semi-flexible molecules also have the option of aligning. There are two cases. The first easily understood case is when the straight shapes are also the low energy shapes. In this case we form either crystals or liquid crystals. The second case is where the low energy shape is some contorted “random walk” shape. Then straightening the molecules in order to pack them in parallel array would raise the energy and not be preferred. Instead the molecules are stuck in their zero or low entropy contorted “random walk” unaligned state [2].

#### A Critical Entropy for Glass Formation

The configurational entropy *S*_c_ for polymers is easily evaluated in the F-H approximation [3–5]. More generally, for non-polymer as well as polymer systems *S*_c_ is defined as the total entropy minus the (proper extrapolation of) vibrational entropy. The volume on the *T*(*P*) line determined from Eq. (1) is not constant; neither is the number of holes in the lattice model. In fact, the configurational entropy can be expressed as a function, *S*_c_(*f*,*n*_0_), of the fraction of flexed bonds, *f*, and the number of holes, *n*_0_. This can be seen clearly from the expression for the partition function *Q*_A_

$$Q_{A}=\sum_{f,n_{0}}\Omega \left(f,n_{0}\right)\exp\left(-\beta E\left(f,n_{0}\right)-\beta PV\right)$$ (2)

where the volume is *V* = *C*(*xn_x_* + *n*_0_), *C* being the volume of a lattice site, *x* the D.P., and *n_x_* the number of polymer molecules. The sum is over all *f*,*n*_0_ such that *Ω* (*f*,*n*_0_) ≥ 1. Since the use of the maximum term is legitimate [4, 5] for this system we have *S*(*f*,*n*_0_) = *k*ln*Ω* (*f*,*n*_0_). The condition *S*_c_(*f*,*n*_0_) = 0, or alternatively *Ω* (*f*,*n*_0_) = 1, divides *f*,*n*_0_ parameter space into the large *f*,*n*_0_ region for which there are large numbers of configurations whose number *Ω* (*f*,*n*_0_) is given by exp(*S*_c_(*f*,*n*_0_)/*k*) for each set of values *f*,*n*_0_ and the small *f*,*n*_0_ region for which there are very few configurations because *S*_c_ = 0 in this region. Both *f* and *n*_0_ vary along the *T*(*P*) transition line which separates the liquid from the glass phase. Below *T*_2_ the values of *f*,*n*_0_ are those which obtain at *T*_2_, *P*_2_ when we cool at constant pressure.

If we vary pressure below the glass temperature *the equilibrium valu*es of both *f* and *n*_0_ change to those values appropriate to the new *T*_2,0_, *P*_2,0_ pair. Although the entropy is zero in the glassy region this only means that lim(*S*_c_/*N*) = 0 as the size of the system *N* → ∞. There can be many allowed configurations below *T*_g_ consistent with this condition and this means that there is sufficient mobility to allow *n*_0_ and *f* to approach their new equilibrium values when pressure is changed. It is important to realize that *n*_0_ is not a constant in the glassy region. Therefore, critical volume cannot be a criterion for glass formation.

Since two independent equations of state (i.e., the *PVT* and the *SVT* equations) completely characterize the thermodynamics, within the accuracy of the lattice model calculation *there can be no other thermodynamic criteria of glass formation other than the vanishing of the configurational entropy, S*_c_ = 0. This important conclusion is supported by arguments for a relation between *S*_c_ and the viscosity *η* (*T*,*P*) [3, 12]. The physical idea for this connection is very clear. If the number of configurations becomes smaller and smaller as we approach the glass temperature from above, flow—which is a moving or jumping from one allowed configuration to another—becomes more and more difficult and consequently the viscosity becomes larger and larger. This suggests that the configurational entropy approaching zero is the universal criterion for glass formation We now quantify the implications of the above statements.

### 1.4 Evaluation of *T*_g_ for Polymers from the *S*_c_ = 0 Condition

If we identify the glass temperature as the point at which the configurational entropy equals zero then

$$S_{c}\left(T_{g},P\right)=0$$ (3)

can be used to determine *T*_g_. We have done this for nine separate classes of experiments on polymers:

1. *T*_g_ vs molecular weight for linear polymers [1, 3].
2. *T*_g_ vs molecular weight for ring polymers [13, 14].
3. *T*_g_ vs copolymer composition [15].
4. *T*_g_ vs blend composition [16, 17].
5. *T*_g_ vs pressure [18, 19].
6. *T*_g_ vs cross-links in rubber [20].
7. *T*_g_ vs strain in rubber [20].
8. Δ*C*_p_ at *T*_g_ for large molecular weight polymers [21].
9. *T*_g_ vs plasticizer (diluent) content [22, 23].

In all cases we obtain reasonable fits to the experimental data. There are several interesting aspects to these comparisons. First, there are essentially no parameter fits to experiment since the model parameters are determined by other independent measurements. In item 1) of the above list we fit to the glass temperature at infinite molecular weight in order to determine the stiffness energy Δ*ε* (one parameter). In 5) we need to assume how the volume of a lattice site varies with pressure (one parameter). the remaining theoretical predictions involve no parameter fits to experiment.

Each class of experiment illustrates a feature of polymer glasses. Item 9) illustrates the colligative-like properties of glasses. The initial glass temperature depression by low molecular weight diluent is predicted [23] to obey the equation

$$\gamma dTN_{g}/dm=-3T_{g}$$ (4)

where *m* is the total mole fraction of diluent expressed in terms of mole fraction of monomers, and *γ* is the number of flexible bonds per monomer. One notices the universal character of the prediction. Item 5) predicts that *T*_g_ vs pressure curves have horizontal asymptotes at high pressure. On the other hand, the free volume theory which assumes that the glass transition occurs when the hole fraction reaches a critically small value (usually 0.025) predicts a vertical asymptote.

In 8) the specific heat change at *T*_g_ for a large molecular weight polymers is given to within 10 % by [21]

$$\Delta C_{p}=Rf\left(1-f\right){\left(\Delta \varepsilon /kT_{g}\right)}^{2}RT_{g}\Delta \alpha \left(4-T_{g}\Delta \alpha /0.06\right)+0.05T_{g}\Delta \alpha C_{p}\left(T_{g}^{-}\right),$$ (5)

where *R* is the universal gas constant, *f* is the fraction of flexed bonds at *T*_g_, Δ*α* is the change in the thermal expansion coefficient as we pass through the glass transition, and $C_{p}\left(T_{g}^{-}\right)$ is the specific heat just below *T*g. Notice that this is a no parameter prediction since *T*_g_, Δ*α*, $C_{p}\left(T_{g}^{-}\right)$ are known from experiment, Δ*ε*/*kT*_g_ is determined from the condition that *S*_c_(*T*_g_) = 0, and *f* is a known function of only Δ*ε*/*kT*_g_ (*f* = 2exp(−Δ*ε*/*kT*_g_)/[1+2exp(−Δ*ε*/*kT*_g_)]. In 2) the glass temperature is predicte*d to rise as we* lower molecular weight of rings in accordance with experiment. This is purely an entropy effect [13, 14] arising from the observation that a ring of molecular weight *x* has more entropy than two rings each of molecular weight *x*/2. Thus a bulk system of the larger rings, since it has the larger entropy, must be cooled further to reach the *S*_c_ = 0 condition which defines *T*_g_. It should be noted that the fits of theory to experiment have all been made with the original Gibbs-Di Marzio theory [4, 5]. We have not needed to adjust the theory to account for new experimental data.

Finally, we should remark that a perfect fit to experiment would require that a) the F-H calculation is perfect. It is not, because the statistics are approximate and because the molecules are modeled imperfectly; b) that the experimental data is excellent, including the use of well characterized polymer material; c) that kinetics have no sensible effect on the comparison with experiment. We would argue that since kinetics are important, perfect accord with experiments would be proving too much. We are predicting the underlying transition temperature *T*_2_, and the relation between *T*_2_ and the experimental *T*_g_ requires further elucidation. We should note that our theory predicts *T*_2_ and not *T*_g_. Since in our equations *T*_2_ appears only in the dimensionalless forms Δ*ε*/*kT*_2_ and *E*_h_/*kT*_2_, if the predictions are correct for *T*_2_ and if *T/T*_g_ depends only on the rate of cooling, then the predictions for *T*_g_ will also be correct. Our good fits to experiments suggest that *T*_2_/*T*_g_ is a constant or *T*_2_ and *T*_g_ are not very different; or some combination of the two. A question we have not examined is “If the criterion for glass formation is *S*_c_ → *S*_c,o_ how well does it predict glass temperatures?” It may suffice for *S*_c,0_ to be small (see below). Mention should also be made of the attempts to predict the glass temperature of a material by simply noting the chemical structure. Figure 10 of Ref. [17] and Fig. 6 of Ref. [24] are remarkable and suggest that further progress can be made. In both of these predictions an entropy criterion is used.

### 1.5 Critique of the Correct Equilibrium Theory of Glasses

An equilibrium theory must satisfy the following criteria:

1. Accurate predictions of thermodynamic quantities without multiplication of parameters.
2. It must explain the ubiquitous nature of glass formation.
3. It must explain why glasses fall out of equilibrium as the glass temperature is approached from above.
4. All predictions must be correct. Since the lattice statistics used for glasses are applicable without change to rigid rod molecules, if the theory is applied to liquid crystals the predictions for this class of materials must also be in accord with experiment.
5. It must provide a foundation for kinetic theory.

We believe we have done reasonably well with regard to criterion 1) as the previous section indicates.

Criterion 2) may be met by first defining the configurational entropy for all materials as the total entropy minus the extrapolation of the vibrational entropy. Any method of evaluating the partition function from first principles which gives the proper equilibrium behavior above *T*_g_ is viable. One would then identify the glass transition as the place where *S*_c_ becomes smaller than some critical value as we cool the system. The following systems need to be examined for their glassy behavior: (a) Polymer glasses, (b) low molecular weight glasses, (c) The classic inorganic glasses, (d) liquid crystals, (e) systems composed of plate-like molecules, (f) spin glasses, (g) plastic crystals, (h) metallic glasses, and (i) gels and thixotropic materials. A common feature of these diverse materials is that they each show frustration-the molecules, or spins, are each prevented from achieving their preferred low energy shape by the interferences of their neighbors. See below.

Under 3) we must be careful not to equate falling out of equilibrium with loss of ergodic behavior. There is a sense in which a system is never ergodic, even at high temperatures. To see this for the case of polymers consider a polymer of *N* monomers which we model as a self-avoiding walk (SAW). An estimate of the number of configurations of one polymer molecule, assuming a cubic lattice is given [25] by 4.86*^N^N*^1/6^ >> 4*^N^* ≈ 10.^0.6^*^N^*. For *N* = 1000 which is a small molecule for polymers, the total number of configurations that can be sampled during the lifetime of the universe which is about 10^10^ years is 10^15^ moves/s × 1000 monomers × 3.6 × 10^7^s/yr × 10^10^ yr = 3.6 × 10^35^. This number is so much smaller than 10^600^ that we see immediately that no system is ever ergodic. Obviously, effective ergodic behavior over some time interval is the relevant concept. By falling out of equilibrium we mean nothing more than that there are certain correlated motions of the molecules that occur with less frequency as we cool the system. At the glass temperature and below they are so rare as to be not measureable. In general one expects that the glass temperature depends on the particular correllated motion being used to monitor it as well as the rate of cooling.

Under 4) above we have the happy circumstance that the same F–H lattice model that was used for glasses also predicts the formation of liquid crystals. As Onsager originally observed [11], the nematic phase of liquid crystals occurs because of the increased difficulty of packing rigid rod molecules together in space as we increase their concentration. Thus, the isotropic to nematic transition in liquid crystals occurs because it is entropy driven—configurational entropy driven. The nematic liquid crystal phase occurs for the same reason as glasses and the correctness of the F–H calculations for liquid crystals argues for their correctness for glasses, and conversely. The transition from random order to parallel alignment for a system of plate-like molecules is also entropy driven [9]. Although the decrease in configurational entropy drives the transition in all three cases the results are somewhat different. Rods and plates have a way out of the packing difficulty; they can align, thereby *increasing* the configurational entropy. Rods lying in parallel with some freedom about the director have a higher configurational entropy than a random packing of rods that is up against its dense packing limit. The molecules forming a glassy material may not have this option. To see this, suppose the lowest energy shape is chosen to be such that the molecules, if they each have this shape (and if we specify that the packing leaves no lattice sites unoccupied), cannot pack in regular array on a lattice; the majority of polymer shapes are of this type [2]. Then alignment at low temperatures is not favored and the material is stuck in its glassy phase.

#### Other Entropy Theories

It is important to improve on theoretical predictions of the equilibrium properties of glass forming materials. One can not expect that the Gibbs-Di Marzio theory which is an elaboration of the Flory-Huggins lattice model is the final word. Improved equations of state would permit more critical tests of the entropy hypothesis to be made. An improved theory should derive the *P*-*V*-*T* and *S*-*V*-*T* equations of state to equal accuracy. A theory that gives a poor *S*-*V*-*T* equation of state is sure to give undue stress to imagined implications of the *P*-*V*-*T* equation of state. The theory must allow the molecules to have shape dependent energies, since these are undoubtedly very important to glassification in polymers. We stress that an improved theory may not show an actual underlying second-order transition as ours does (there may be a rounding), but it should approximate one.

Two theories that include the effects of stiffness energy are those of Gujrati and Goldstein [26] and of Milchev [27]. We accept that Gujrati has calculated a rigorous lower bound to the entropy for a two dimensional square lattice. This means that we must modify our criterion of glassification, viz. *S*c → 0, to something else.

We do not accept the Milchev criticism [27] because his formula does not show the phenomenon of frustration which we take to be an essential feature of glassification and, for rigid rods, an essential feature driving the isotropic phase towards the nematic phase. Specifically, in the Milchev theory individual polymer chains are never prevented from achieving their Boltzmann distribution of shapes which are given in the simple nearest neighbor model by *f* = (*z* − 2)exp(−/*kT*)/[1 + (*z* − 2)exp(−Δ*ε/kT*)] where *z* is the coordination number of the lattice. In the Gibbs-Di Marzio model this distribution is realized above *T*_2_, but at lower temperatures each chain is frustrated by its neighbors from achieving its Boltzmann distribution of shapes. Instead, the distribution that existed at *T*_2_, *P*_2_ persists as we lower the temperature at constant *P*_2_. The number of holes also remains constant below *T*_2_ while in the Milchev theory it continues to decrease. The fact that experimentally the volume versus temperature curve for a glass parallels the volume versus temperature curve for a crystal supports the view that the number of holes is constant below *T*g. It must be mentioned however that some computer calculations exist that support the Milchev formula [28].

### 1.6 Modification of the *S*_c_ = 0 Criterion to *S*_c_ = *S*_c,0_

One reason for the configurational entropy to be somewhat greater than zero at the glass transition has to do with the concept of “percolation of frustration” as a criterion of glass formation. As an entree to this problem we express the configurational entropy as a function *S*_c_(*f*,*n*_0_) of the two order parameters *f* (the fraction of flexed bonds) and *n*_0_ (the number of empty lattice sites). The equation

$$S_{c}\left(f,n_{0}\right)=k\ln\Omega \left(f,n_{0}\right)=0$$ (6)

divides *f*,*n*_0_ parameter space into two regions, the large *f*,*n*_0_ region being the liquid region and the line defined by Eq. 6 gives *f*,*n*_0_ values appropriate to the glass. Because there are spatio-temporal fluctuations in *f* and *n*_0_, if we are in the liquid region just above the glass region there will be “clusters” of polymer for which the *f*,*n*_0_ values are appropriate to the glassy state, and clusters for which the *f*,*n*_0_ values are appropriate to the liquid state. As we lower the temperature these glass-like clusters grow until they span the space or percolate. However, as is characteristic for percolation [29] there will be pockets of liquid-like clusters (regions of material for which the f,*n*_0_ values are appropriate to the liquid phase). The glass temperature would be defined as the highest temperature for which there is percolation of the glass-like structures. Because of the existence of the liquid-like pockets this *T*_g_ would correspond to a configurational entropy somewhat greater than zero. This percolation view of glasses receives support from experiments which show anomalously high mobility as the temperature is decreased through the glass transition [30]. The unexpectedly high mobility seems to arise from pockets of fluid dispersed in a glassy matrix.

**Fig. 1:**
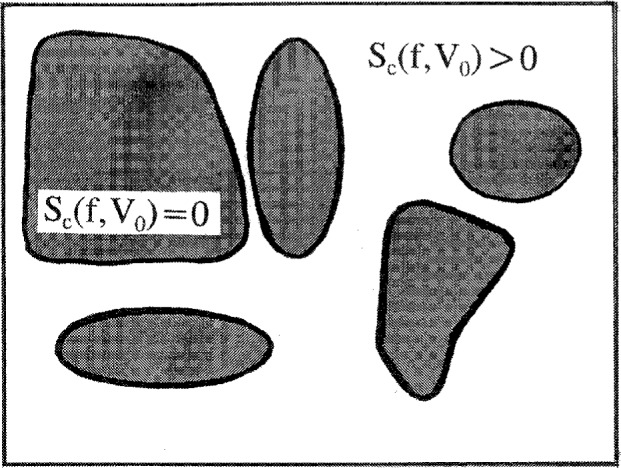
In the lattice model version of the entropy theory of glasses there are two global order parameters: *f*, the fraction of bonds flexed and *V*_0_, the fraction of empty lattice sites. The glass transition occurs when these quantities which decrease with decreasing temperature and increasing pressure have values which make the configurational entropy *S*_c_(*f, V*_0_) equal to zero. However, since *f* and *V*_0_ have spatial and temporal fluctuations we know that as we approach the glass from above there will form pockets of material for which the order parameters are appropriate to the glass imbedded in a sea of liquid with regions of *f* and *V*_0_ appropriate to the liquid state. Percolation theory tells us that when these pockets connect up into an infinite cluster there will remain pockets of liquid. If we define the thermodynamic glass transition as the percolation point then the configurational entropy will be greater than zero at the transition temperature.

The percolation argument can be quantified by allowing *f* and *n*_0_ to vary in space. Consider the following diagram (see also Fig. 1).

**Figure:**
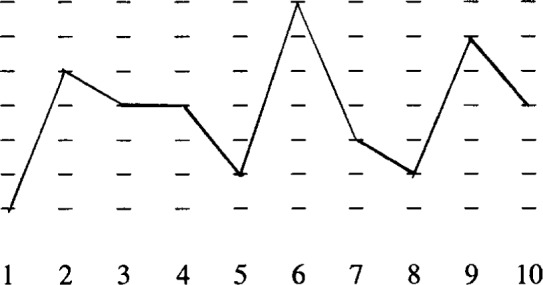


Here the numbers indicate positions in space (cells) and the dashes above a given number denote values of *f*(***r***),*n*_0_(***r***). The line connecting the 10 places is one particular enumeration of *f*,*n*_0_ in space. Each cell contains *n* lattice sites and there are *N*/*n* cells where *N* is the total number of sites on the lattice. To obtain the total partition function we take the product of the partition functions for each cell. Thus,

Q=Πq(r) (7)

where the product is over space, and

$$q\left(r\right)=\Sigma \omega \left(f\left(r\right),n_{0}\right)\exp\left(-\beta u\left(f\left(r\right),n_{0}\right)\right)$$ (8)

where the summation is over all *permitted* values of *f*,*n*_0_ at the cell labeled by ***r*** values on the high side of the line *S*(*f*,*n*_0_) = 0) in *f*,*n*_0_ parameter space. It is evident that all possible *f*(**r**), *n*_0_(**r**) values are thereby accommodated. It is easy to see that the value of entropy calculated from this procedure is larger than that calculated from Eq. (2) for the simple reason that *Q* includes a sum over many paths while *Q*_A_ does not include such a sum. This argument suggests that we should replace the *S*_c_ → 0 criterion for the occurrence of the glass transition by the less stringent criterion

$$S_{c}\left(T_{2},P_{2}\right)\to S_{c,0}$$ (9)

where *S*_c,0_ is some critical value of the configurational entropy. This is in accord both with the ideas of percolation [29] and with the experimental observation that small pockets of polymer within the glassy region can show kinetic behavior that is not simply vibrational behavior (crankshaft motion [31], for example). *S*_c,0_ would be that value of entropy for which the glassy regions first percolate as the temperature is lowered.

### 1.7 Qualitative Insights Into the Kinetics of Glass Formation Arising From the *S*_c_ → 0 Criterion

We wish to determine how the kinetics relates to the configurational entropy *S*_c_ as we cool our system. Now, how one speaks about entropy depends on the kind of ensemble one is working with. We have used [4, 5] the Canonical Ensemble for which *S*_c_(*T*)/*k* = Σ*f_i_* ln*f_i_* but because the system size is large we can also write *S*_c_(*T*)/*k* = ln*W*(*T*) where *W*(*T*) is the number of configurations whose energy is the average energy *E*(*T*) determined from the Canonical ensemble. This enables us to speak in terms of the microcanonical ensemble.

Thus, as we lower the temperature the number of configurations *W* decreases so that they are farther apart in phase space [that part of phase space for which the total energy is *E*(*T*)]. The process of diffusion as well as the process of flow can be viewed as a jumping out of a deep well, a subsequent wandering about the phase space between deep wells and a dropping into a deep well different from that from which it had exited. The process then repeats itself. Obviously this process becomes more infrequent at lower temperatures resulting in increased viscosity and decreased diffusion. There are several reasons for this. First, the deeper the well the longer the time to escape it—wells are effectively deeper as we lower temperature; second, the further apart the wells are the more time it takes for a phase point to wander from one well to another; third, the further apart the wells the larger the probability that the wandering phase point will fall back into the well it has just escaped resulting in no net flow. See Appendix A for a discussion of this effect. The picture we are using is a variant of the trapping model with the difference that instead of an atom or an electron being trapped we are trapping the phase point (or configuration point [32]). In Sec. 2 we shall quantify these ideas.

### 1.8 An Insight From the Fluctuation Dissipation Theorem

Whenever there is a thermodynamic phase transition the fluctuation-dissipation (F–D) theorem [33] suggests that dissipative quantities have the same discontinuities as the underlying thermodynamic phase transition: A simple example of a F–D theorem is the Green-Kubo [34] relation

$$D=\left(1/3\right)\int_{\;}^{\infty }\langle v\left(0\right)\cdot v\left(t\right)\rangle \;dt$$ (10a)

which relates the diffusion coefficient *D* to the autocorrelation function of the particle velocity ***v***. More generally the F–D theorem relates ***χ*** (defined as the response of a material at (***r***,*t*) arising from an impulsive force at (***0***,0)) to the correlation in fluctuation at these two space-time points [25]. Since the fluctuations of a system at equilibrium show a discontinuity of the same character as the thermodynamic extensive variables, so do also the dissipative quantities. Thus, for a system undergoing a first-order liquid to crystal transition the viscosity *η* (*ω, T, P*) will show a discontinuity as a function of *T, P* since the volume and entropy do. Similarly, for a system undergoing a second-order transition we can expect that the viscosity will show discontinuities in slope since the volume and entropy do (see later in this paper however). There are many examples in the literature of dissipative quantities such as viscosity, diffusion coefficient, electrical conductivity, particle conductivity and thermal conductivity which show breaks as a function of temperature as we pass through the glass transition. However, it is also true that a genuine falling out of equilibrium will also cause the same kind of behavior. It is uncertain how one distinguishes between the two effects. Movement of the transition point as a function of the time-scale of the experiment seems not to be a distinguishing characteristic since this happens also for systems known to have genuine first-order transitions-supercooling being an obvious example.

More generally the frequncy dependent diffusion coefficient is given by

$$D\left(\omega ,T\right)=\int_{0}^{\infty }\exp\left(+i\omega t\right)\langle v\left(0\right)v\left(t\right)\rangle \;dt.$$ (10b)

## 2. Kinetic Theory of Glasses

### 2.1 A Remark on the Topology of Phase Space

The potential energy surface of a liquid *E*′(.. *q_j_*..) appears in the partition function *Q*

$$\begin{array}{l} \;\;\;\;\;\;\;Q=\int_{\left\{..q_{j},p_{j}..\right\}}^{\;}\exp(-(K\left(\left\{..q_{j},p_{j}..\right\}\right)\\ \;\;\;\;\;\;\;\;\;\;\;\;+E^{\prime }\left(..q_{j}..\right))/kT)\Pi dq_{j}\;\Pi dp_{j}\\ =\Lambda^{3N}\int_{\left\{..q_{j}..\right\}}^{\;}\exp\left(-E\left(\left\{..q_{j}..\right\}\right)/kT\right)\Pi dq_{j}, \end{array}$$ (11)

where *q*_j_, *p_j_* are the generalized position and momentum coordinates of the *N* particles, *K* is the kinetic energy, *E*′ is the potential energy and *Λ* the thermal wavelength. Since the kinetic energy is quadratic in *p_i_* the integration over *p_i_* is straightfoward. In polymers, even if *E′* is pairwise additive, *E* is not [35] because the coefficients of the quadratic terms in *K* are in general dependent on *q_i_*. The simplification of Eq. (11) allows us to work exclusively in configuration space. This is generally represented as a multi-well potential energy surface. As one approaches *T*_g_ from above the wells effectively become very deep because of the 1/*kT* term. One then talks about flow as a motion from one deep well to another deep well via the higher energy continuum.

Here, however, we wish to emphasize a different aspect of the phase space topology. Consider the configuration space of *N* identical noninteracting hard point particles on a line of length *L*. The partition function is given by

$$Q=L^{N}/N!.$$ (12)

The volume of configuration space for this system is given by *L^N^*. Now consider the case where the particles each have a diameter *d*. The partition function is

$$Q_{d}={\left(L-Nd\right)}^{N}/N!$$ (13)

The ratio of the two phase space volumes is given by

$$\begin{array}{c} Q_{d}/Q={\left(1-Nd/L\right)}^{N}\leq \exp\left(-N^{2}d/L\right)\\ =\exp\left(-\phi N\right), \end{array}$$ (14)

where *ϕ*, being the volume fraction occupied by the particles, is on the order of 1. Since *N* is on the order of Avogadro’s number we see that the fraction of the volume of phase space occupied by the extended particles is infinitesimally small relative to the unconstrained particles. Based on this picture a point in configuration space wanders on the finest of gossamer threads [36] which pervade the *N*-dimensional hypercube of phase space as a fine network whose total volume is an infinitesimal fraction of *L^N^*. The application to glasses is in the observation that as we lower *T* the effective value of d increases, resulting in even fewer and finer gossamer threads for the phase point to travel on. Thus, not only are the number of paths (threads) between two phase points fewer as we decrease *T* but also as one traverses a given thread the potential energy minima are effectively deeper and the barriers effectively higher.

The above discussion serves to show how important it is to know how the deep wells are connected to other wells. In order to solve this problem we need to construct a model of the topology of configuration space and to calculate the transition rates for jumping from well to well within this model.

### 2.2 Detailed Balance Makes A Significant Statement Concerning the Kinetics of Glasses

Boltzmann’s law gives exp(− *E_i_*/*kT*) as the fraction of time that a system spends in state *i* but it does not say how often the system jumps from state *i* to state *j*. To determine this we use the principle of detailed balance in the form

$$\begin{array}{l} N_{i}\alpha_{ij}=N_{j}\alpha_{ji},\alpha_{ij}/\alpha_{ji}=N_{j}/N_{i}\\ \;\;=\exp\left(-\left[E_{j}-E_{i}\right]/kT\right), \end{array}$$ (15)

where *α_ij_* is the rate of jumping from state *i* to *j* and *N_i_* is the fraction of time a system spends in state *i*. In using Eq. (15) one must first decide how the energy is apportioned into forward and backward transitions. For deep wells it is sensible to assume that all of the barrier is in preventing the phase point from jumping out of the well. It does this at a rate given by 1/*τ* where *τ* is the average time to exit the well. If we also recognize that the probability of jumping out of the well is exponential in time [37] we have

$$\begin{array}{l} P\left(t,\tau \right)=\tau^{-1}\;\exp\left(-t/\tau \right)\;\text{and}\;\\ \tau^{-1}=b_{ij}\;\exp\left(-\left[E_{j}-E_{i}\right]/kT\right), \end{array}$$ (16)

where *P* is the probability density of exiting the well at time *t*. It is imagined that once the phase point has escaped the well it wanders around in the configurational sea of the high energy region of phase space until it falls into a low lying well, starting the flow process all over again. This configurational sea consists of many shallow energy wells, so it is expected that jumping out of the deep wells are the rate determining steps.

### 2.3 Diagrams for Our Minimal Models and Their Associated Equations

**Fig. 2:**
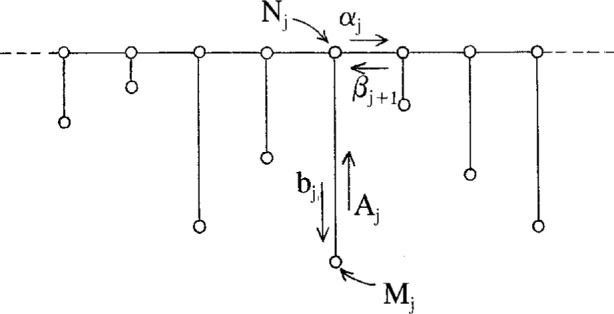
Our minimal model for describing the kinetics of glasses. The points are points in configuration space and the connecting lines represent allowed transitions between points. The horizontal lines with rates *α*_j_ for traveling to the right and *β_j_*_+ 1_ for traveling to the left represent travel of the configuration point among the “configurational sea” of shallow wells. The vertical lines connect the “configurational sea” to the deep wells, the length of the vertical line being proportional to the potential energy depth of the well. The rate of escape from the deep wells is *A_j_* and rate of capture is *b*. When the configuration point is in a deep well there is no motion; motion occurs only when the configuration point is cruising the configurational sea of shallow wells. This trapping model allows us to infer an important contribution to the complex viscosity η*(*ω*< *T*), the diffusion coefficient *D*(*ω,T*) and dielectric response *ε* (*ω, T*).

**Fig. 3:**
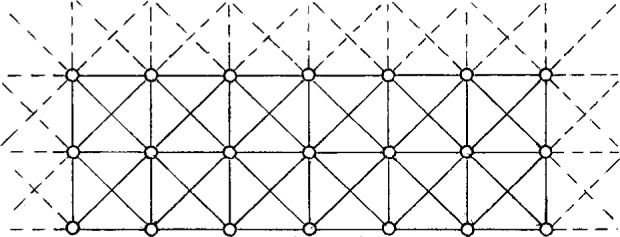
The set of horizontal lines and their connecting points in Fig. 2 really represent the vastly more complicated diagram of Fig. 3. The occupation probability *N_j_* of Fig. 2 is really the sum Σ*N_j,i_* of Fig. 3 and the *α* and *β* of Fig. 2 are compounded from the rate constants of Fig. 3. The net result is that the *α* and *β* are much larger than *b* and *A* in Fig. 2.

The diagram for our primary minimal model is displayed in Fig. 2. This diagram is a contraction of a vastly more complicated diagram but we believe it retains the essential features of glassy behavior. The points on the upper line represent the multitude of shallow wells while the horizontal lines connecting these points represent the transition rates between these wells. This set of horizontal lines and points represent the vastly more complicated diagram of Fig. 3. At high temperatures this “configurational sea” of shallow wells is where all the action is; The configuration point jumps rapidly from well to well. The occupation number *N_j_* for well *j* in Fig. 2 is really the sum of the occupation numbers vertically above it in Fig. 3, and the transition rate for the horizontal bonds of Fig. 2 is compounded from those of Fig. 3.

The lower points represent the deep wells. Our view of what happens is as follows. At low temperatures the configuration point is in one of the lower wells. After a long period of time it jumps out and wanders about the configurational sea of upper wells until it falls into a low lying well. It then stays in this well for another long period of time until it jumps out repeating the process, and so on. The situation at high temperatures as described in the preceding paragraph is very different. There are so few deep wells relative to the number of upper wells that they are unimportant; all the motion is jumping among the upper wells. The rate constants for jumping out of these lower wells are much much smaller than that for jumping back down into the well and than those for traveling horizontally. By adjusting the ratio of the rate constant for falling back into the deep well to that for traveling horizontally we can control the accessibility that the configuration point in the configurational sea has for the deep wells.

**Fig. 4:**
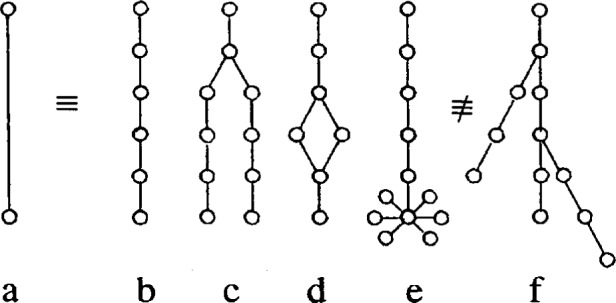
The vertical lines in Fig. 2 represent many possible paths in configuration space leading to the deep wells. In Fig. 4 we have listed five possible paths to deep wells, or equivalently ways to decorate each of the vertical lines of Fig. 2. It can be shown that diagrams b, c, d, and e are equivalent to a. Thus, the diagram of Fig. 2 really represents vastly more complicated diagrams formed by decorating Fig. 2 by the diagrams of Figs. 3 and 4. Thus, the equations in the text describing Fig. 2 have a wider applicability. However incorporation of Fig. 4f would require us to replace *A_j_* by a memory kernel in the equations describing the diagram. See text.

The length of the vertical line connecting the deep well to the upper well(s) is proportional to the well depth. These vertical lines represent many possible paths in configuration space leading to the deep well. In Fig. 4 we have listed some of the possibilities. Figs. 4c, 4d, 4e can each be shown to be equivalent to Fig. 4b. To see this, one writes down by the methods of Ref. [38] the set of equations corresponding to a given figure and then one shows that they can be transformed to the set of equations describing Fig. 4b. The rate constants in the transformed set of equations are such that the occupation probabilities at each level are the same as those in the untransformed figure.

It can also be shown using the methods developed previously [38] that Fig. 4b is equivalent to Fig. 4a. Specifically, one can choose rate constants for the upward and downward steps in Fig. 4a that are compounded from those of Fig. 4b in such a way that the occupation of the bottom well in Fig. 4a equals the sum of those in Fig. 4b in both the equilibrium and the flux determined [38] steady state solutions.

However, Fig. 4f has a different structure entirely. In a descent of the configuration point from the configurational sea into this structure it can get hung up in a branch so that it may take a long time for it to reach equilibrium. The other figures all equilibrate rather quickly.

The results of this paper will allow us to conclude that although Fig. 3 is rather simple it does catch the essential features of glassification.

The Master Equations describing the minimal model of Fig. 2 are given by the simple set of equations

$$\begin{array}{c} dN_{1}/dt=-\left(\alpha_{1}N_{1}-\beta_{2}N_{2}\right)-b_{1}N_{1}+A_{1}M_{1}\\ dN_{j}/dt=\left(\alpha_{j-1}N_{j-1}-\beta_{j}N_{j}\right)-\left(\alpha_{j}N_{j}-\beta_{j+1}N_{j+1}\right)\\ -b_{j}N_{j}+A_{j}M_{j} \end{array}$$ (17a)

$$dM_{j}/dt=+b_{j}N_{j}-A_{j}M_{j}$$ (17b)

where the Greek symbol rate constants denote stepping to the right (*α*) or left (*β*) and the Roman symbol rate constants denote stepping down (*b*) or up (*A*).

#### 2.3.1 Going from Phase Space to Configuration Space to Real Space

We have already shown in Sec. 2.1 that one can integrate over all the momentum variables of phase space so that we deal only with position variables (configuration space). We would like to go further and deal with the smallest number of position variables possible. We begin by supposing that there are two separate noninteracting regions of space each with their own master equations

$$df_{j}/dt=\Sigma f_{r}\alpha_{rj}-\Sigma f_{j}\alpha_{jr}$$ (18a)

$$d{f^{\prime }}_{k}/dt=\Sigma {f^{\prime }}_{s}{\alpha^{\prime }}_{sk}-\Sigma {f^{\prime }}_{k}{\alpha^{\prime }}_{ks}$$ (18b)

where *f_j_* is the fraction of systems in state *j* and *α_j_*_r_ is the rate of jumping from state *j* to *r*. Multiplying the first equation by *f*′*_k_* and the second by *f_j_* we obtain

$$\begin{array}{c} d\left({f^{\prime }}_{k}f_{j}\right)/dt=\Sigma {f^{\prime }}_{k}f_{r}\alpha_{rj}-\Sigma {f^{\prime }}_{k}f_{j}\alpha_{jr}+\Sigma f_{j}{f^{\prime }}_{s}{\alpha^{\prime }}_{sk}-\Sigma f_{j}{f^{\prime }}_{k}{\alpha^{\prime }}_{ks}\\ =\Sigma \;\Sigma f_{r}{f^{\prime }}_{s}\left(\alpha_{rj}\delta_{sk}+{\alpha^{\prime }}_{sk}\delta_{rj}\right)-\Sigma \;\Sigma f_{j}{f^{\prime }}_{k}\left(\alpha_{jr}\delta_{sk}+{\alpha^{\prime }}_{ks}\delta_{rj}\right)\\ =\Sigma \;\Sigma \left(f_{r}{f^{\prime }}_{s}\right)A_{rs;jk}-\Sigma \;\Sigma \left(f_{r}{f^{\prime }}_{k}\right)A_{jk;rs}. \end{array}$$ (19)

Or, if we relabel the indices so that *ℓ* ≡ (*r*,*s*) and *i* ≡ (*j*,*k*) then we obtain

$$dN_{i}/dt=\Sigma N_{l}\;A_{li}-\Sigma N_{i}A_{il}$$ (20)

which is the master equation for the composite system. Notice that the complexion, *N_i_*, of the composite system is the product of the complexions, *f_j_*, of the individual systems, but the composite transition coefficients are sums of the individual transition coefficients. These results are readily generalized by the process of induction to a system consisting of any number of subsystems, the only condition being that the subsystems do not interact with each other. We again see that the complexion of the composite system is the product of the complexions of the individual systems, but the composite transition coefficients are sums of the individual transition coefficients.

Thus, if we could find a smallest set of independently interacting molecules we will have simplified our problem considerably. Fortunately there is a confluence of intuition and experiment that suggest that this can be done. First, what is happening at point a cannot be influenced by what is happening at point b provided that the two points are sufficiently far apart. So, there is a smallest size. Second, this size seems to be very small indeed. Stillinger, on the basis of computer modeling and other considerations has concluded [39] that the number of molecules involved in the basic diffusion step is on the order of several molecules for simple van der Waals systems. Perhaps a local density decrease allows a molecule to jump out of a cage, or perhaps two molecules interchange, resulting in a net flow.

As a result of these considerations we can maintain that the *N_i_, M_i_* of Eqs. (17) refer not only to configuration space, but also to particles or quasiparticles in our 3-d space. A connection is thus made between the trapping model of Di Marzio and Sanchez [32] who trapped the configuration point and the trapping model of Odagaki et al. [40] who trapped atoms. Of course trapping atoms implies trapping the configuration point and conversely. The context of the discussion easily determines what kind of particle or quasiparticle is being trapped.

Equations (17) can be transformed into a continuum version by using

$$N_{j}\left(t\right)\to N\left(t,x\right),N_{j+1}\left(t\right)\to N\left(t,x+\Delta x\right),M_{j}\to M\left(t,x\right),\text{etc}.$$

$$\alpha_{j}\to \alpha \left(x\right),\beta_{j}\to \beta \left(x\right),\alpha_{j+1}\to \alpha \left(x+\Delta x\right),\text{etc}.$$ (21)

we obtain

$$\partial N/\partial t=\partial^{2}\left(DN\right)/\partial x^{2}-\partial \left(vN\right)/\partial x-bN+AN$$ (22a)

∂M/∂t=+bN−AM, (22b)

where

$$D={\left(\Delta x\right)}^{2}\left(\alpha +\beta \right)/2\;\text{and}\;v=\left(\Delta x\right)\left(\alpha -\beta \right).$$ (23)

*D, v, b*, and *A* can all be position dependent.

The rate constants are determined as follows. From Eq. (15) we have

$$A_{j}=b_{j}\;\exp\left(-\left|E_{j}\right|/kT\right),b_{j}=b,$$ (24)

where *E*_j_ is the depth of the well. We argue that the energy appears only as a barrier restricting the escape from the wells-there is no attraction of the phase point into a well. The *b_j_* are also all chosen to be equal because we can think of nothing that distinguishes them from each other. Allowing the *α_j_* to be different from the *β_j_* accounts for a drifting of the phase point towards a region of phase space. This should be useful if we impose an external field. If we assume no *x* dependence for *α* and *β* then *D* and *v* are constants and the *∂*^2^*DN*/*∂x*^2^ term is the ordinary diffusion term. Our Equations now read

$$\begin{array}{l} \partial N\left(t,x\right)/\partial t=D\partial^{2}N\left(t,x\right)/\partial x^{2}\\ -v\partial N\left(t,x\right)/\partial x-bN\left(t,x\right)+A\left(x\right)N\left(t,x\right) \end{array}$$ (25a)

∂M(t,x)/∂t=bN(t,x)−A(x)M(t,x), (25b)

where we have written all *t, x* dependencies explicitly.

Since *α* and *β* are much greater than b we know that after jumping out of a low lying well the phase point will travel extensively horizontally before being captured by a deep well. Since *b* does not depend on *x* and is not a function of well depth the rate of filling the wells is random. Thus the horizontal distribution of well depths which we assume to be random along the chain (see Fig. 2) is unimportant. If *W*(*E*) is the number of wells of depth *E* then they are filled with a rate proportional to *W*(*E*). Over a large period of time the escaping from wells is determined by both *W*(*E*) and the rate of escape (exp(− *β* |*E* |)) from individual wells. This allows us to replace the distribution of wells by wells of one depth. In this case Fig. 2 becomes simplified even further so that the vertical lines have the same length. The equations now can be simply solved since *A* now has no *x* dependence. Using the method of moments on Eqs. (25) we find

$$\begin{array}{l} d\langle N\rangle /dt=-b\langle N\rangle +A\langle M\rangle \\ d\langle M\rangle /dt=b\langle N\rangle -A\langle M\rangle \end{array}$$ (26a)

$$\begin{array}{l} d\langle xN\rangle /dt=v\langle N\rangle -b\langle xN\rangle +A\langle xN\rangle \\ d\langle xM\rangle /dt=b\langle xN\rangle -A\langle xM\rangle \end{array}$$ (26b)

$$\begin{array}{c} d\langle x^{2}N\rangle /dt=2D\langle N\rangle +2v\langle xN\rangle \\ \;-b\langle x^{2}N\rangle +A\langle x^{2}M\rangle \\ d\langle x^{2}M\rangle /dt=b\langle x^{2}N\rangle -A\langle x^{2}M\rangle . \end{array}$$ (26c)

The nice thing about these equations is that we can solve the *n*th pair of equations for the nth order moments in terms of the lower order sets. We will exploit this fact in the next section.

Finally, considering only the sequence in time of the occupation of the deep wells by the configuration point, with *b_j_* = *b*, Σ*N_j_* = *nN*, and assuming that horizontal motion is so fast that *N_j_* = *N*, the sum over the *N_j_* in Eq. (17a) yields,

$$ndN/dt=-nbN+\Sigma A_{j}M_{j}$$ (27a)

$$dM_{j}/dt=bN+A_{j}M_{j}$$ (27b)

**Fig. 5:**
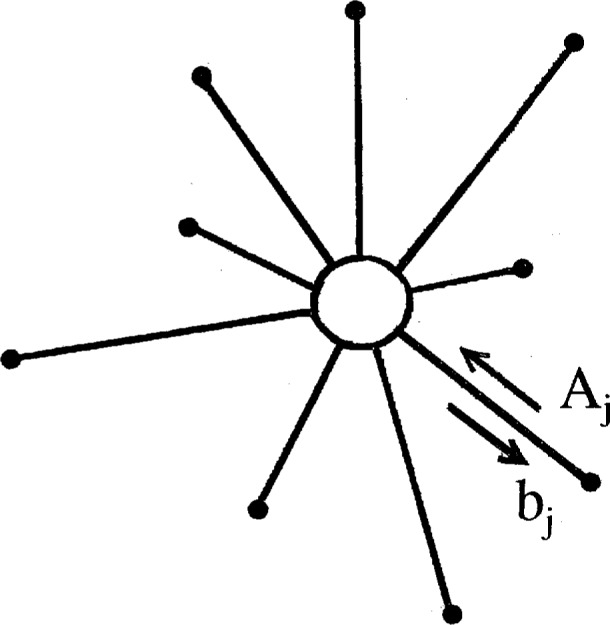
If the *α* and *β* of Fig. 2 are very much larger than the *b* and *A* then we can argue that the configuration point running about in the “configurational sea” sees an unbiased statistical sample of the wells before falling out of the “configurational sea” into any one of them. Thus if we are interested only in the sequence in time of occupation of the wells by the configuration point the diagram of Fig. 5 suffices. In the text the simplified equations describing Fig. 5 are obtained.

Here the total number of shallow wells is n. Figure 5 displays the diagram associated with these equations.

One notes that Eqs. (17), (22), and (25) are very similar to equations arising in modeling chromatography [41]. In that case the diffusion and drift terms model the behavior of the eluting material as it travels along in the mobile phase, *N*(*t, x*) being the amount of material in the mobile phase, while *M*(*t, x*) is the amount of material adsorbed on the adjacent surface or in pores [42].

Our minimal models are all now well defined and deriving their implications is merely a matter of mathematics, albeit sometimes very difficult mathematics. The remaining conceptual problem, to which we now turn, is to relate the solution of these minimal models to the frequency and temperture dependent complex viscosity *η* *(*ω*< *T*), diffusion coefficient *D*(*ω*< *T*) and dielectric response *ε* (*ω*< *T*).

### 2.4 Insights From Our Minimal Models: Derivation of *D*(0, *T*) And *η* (0, *T*)

#### 2.4.1 The Diffusion Coefficient *D*(0, *T*) When All Wells Have the Same Depth

Equations 26 are easily solved for the moments. After some labor, with obvious assumptions on the initial conditions we obtain to first order in the drift velocity

〈x〉≡〈x(N+M)〉/〈(N+M)〉=(A/(b+A))vt (28)

$$\begin{array}{c} \langle {\left(x-\langle x\rangle \right)}^{2}\rangle \equiv \langle {\left(x-\langle x\rangle \right)}^{2}\left(N+M\right)\rangle /\langle \left(N+M\right)\rangle \\ =\left(A/\left(b+A\right)\right)2Dt. \end{array}$$ (29)

Notice that the diffusion coefficient is diminished by the factor *A*/(*b* + *A*) (because from Eq. (24) *A*/*b* = exp(− |*E_j_* |/*kT*) and the wells are deep we will ignore the *A* in the denominator of *A*/(*b* + *A*)). These equations have the obvious interpretation that everything, both drift and diffusion, is being slowed down by the factor *A*/*b* which is the ratio of jump rates. As long as the particle is in a deep well there is no activity. Any resulting activity is proportional to the rate of escape, exp(− |*E_j_*|/*kT*), from the deep wells.

**Fig. 6:**
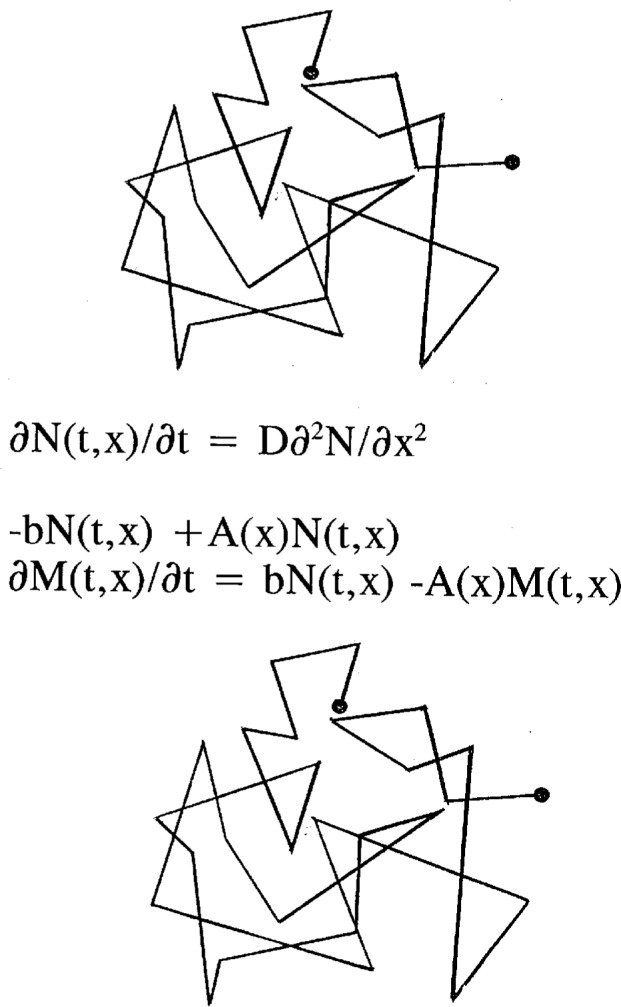
a) In the probabilistic interpretation of the diffusion equation *∂C*/*∂t* = *D*∇^2^*C* the Green’s function represents a random walk with no pausing time between steps of the random walk. b) In the probabilistic interpretation of the equations describing our minimal model the trapping in deep wells corresponds to a pausing time between steps of the random walk. The spatial aspects of the walks are identical in both cases.

We now seek to further interpret this result. The ordinary diffusion equation without sinks (*∂N*/*∂t* = *D∂*^2^*N*/*∂x*^2^) has as its Green’s function the Gaussian distribution (4*πDt*)^1/2^exp(− *x*^2^/4*Dt*). In the probabilistic formulation of the diffusion equation this Green’s function has the physical interpretation of representing a random walk as in Fig. 6a. There is no pausing between steps of the random walk. However, the equations of our minimal models have the interpretation that when the particle is in a deep well there is no motion until, after a long time the particle escapes the well. Thus, in the probabilistic interpretation of our minimal models our physical process is represented by a random walk with a pausing time between steps. The steps themselves correspond to the horizontal motion characterized by the diffusion constant *D* while the pausing corresponds to the time spent in the deep wells. Thus, the effective diffusion coefficient is

$$\begin{array}{l} D_{e\mathrm{ff}}\left(0,T\right)={\left(\Delta x\right)}^{2}/2{\left(\Delta t\right)}_{\mathrm{eff}}\\ =\left(A/b\right)D=(\left(A/b\right){\left(\Delta x\right)}^{2}/2\Delta t\\ =\exp\left(-\left|E_{j}\right|/kT\right){\left(\Delta x\right)}^{2}/2\Delta t. \end{array}$$ (30)

#### 2.4.2 The Viscosity *η* (0, *T*) When All Wells Are of the Same Depth

In Eq. (30) we have taken the view that the paths traversed in configuration space are the same for both the case of pure diffusion and that of diffusion with traps (See Fig. 6). This means that the only difference between the two cases is the time to take each step. For diffusion with traps we write

$$\Delta t_{\mathrm{eff}}=\Delta t+\Delta t_{\mathrm{well}},\Delta t<<\Delta t_{\mathrm{well}}.$$ (31)

where Δ*t*_well_ is the time spent in the traps between jumps, while Δ*t* is the time spent traversing the path in the configurational sea (the time spent between jumping out of one well and falling into the next well). Since viscosity is inverse to diffusion we will assume that the viscosity is proportional to the average time spent in the deep wells. This notion is verified in Appendix B. Thus,

$$\eta \left(0,T\right)/B\propto \langle t\rangle =\int_{0}^{\infty }tP\left(t,T\right)dt$$ (32)

where *P*(*t*,*T*) is the normalized probability density that the configuration point escapes the well at time *t*.

When all the wells are of the same depth *P*(*t*,*T*) is easily calculated. The probability ***ψ***(*t*,*T*) of the particle being in the well at time *t* is

$$\begin{array}{r} \psi \left(t,T\right)=\exp\left(-bt\exp\left(\left|E\right|/kT\right)\right)\\ =\exp\left(-t/\tau \right);\tau =b^{-1}\;\exp\left(+\left|E\right|/kT\right) \end{array}$$ (33)

and the probability density *P*(*t, T*) for exiting the well at time *t* is

$$P\left(t,T\right)=\partial \psi /\partial t=\tau^{-1}\;\exp\left(-t/\tau \right).$$ (34)

The exponential approximation for ***ψ*** is a good one. To see why consider the configuration point in a well. It decays exponentially initially. This can be seen by solving the generic matrix equation to which the set 27 belongs

dN/dt=AN (35)

where *N* is the set (*N_j_, M_j_*) and *A* is the matrix of Eqs. (27). We obtain [43]

N=exp(+At)N(0) (36)

and if we begin with one particle in one well we see that for small t we have our exponential decay. But when the particle jumps out of this well the chance that it comes back into the same well is very small since there are so many other wells. Thus, we are confident of our assumed form [(Eq. 34)]. However, it is stressed that Eqs. (27) should be solved rigorously to bolster the argument.

Equation (34) when substituted into Eq. (32) gives

$$\begin{array}{c} \eta \left(0,T\right)/B\propto \langle t\rangle =\int_{0}^{\infty }tP\left(t,T\right)dt\\ =\int_{0}^{\infty }t\tau^{-1}\;\exp\left(-t/\tau \right)dt=\tau \end{array}$$ (37)

which was to be expected.

#### 2.4.3 *η* (0, *T*) When the Wells Are of Different Depths

However, solving the problem where the deep wells are all the same depth is not the same as solving the problem for glasses since glasses have a distribution of well depths. We need to evaluate *P*(*t, T*) for this latter case and also calculate a new effective diffusion coefficient. *P*(*t, T*) is exactly calculable from Eqs. (27) since in the probabilistic interpretation the configuration point jumps from well to well, and there is no Δ*x* involved in Eqs. (27). A configuration point in a well of depth *E* sees only the barrier and therefore the probability that it be in the well at time *t* is given by Eq. (33). Let *W*(*E*) be the weight distribution for wells of depth *E*. Notice from Eq. (37) that *∫W*(*E*)*P*(*t, T*)d*t* = *b*^−1^*W*(*E*)exp(|*E_j_*/*kT*) which states that the time spent in wells of level *E* is given by the Boltzmann factor weighted by the degeneracy factor *W*(*E*). This is in perfect accord with the ergodic theorem. An estimate of the relaxation function *p*(*t, T*) describing the exiting from wells can now be made by weighting the distribution function *P*(*t*,*T*) (see Eq. (34)) for the occupation of the well of depth *E* by the weighting function *W*(*E*).

$$\begin{array}{c} p\left(t,T\right)=\int W\left(E\right)P\left(t,T\right)dE/\int W\left(E\right)dE,\\ =\int W^{\prime }\left(E\right)P\left(t,E\right)dE \end{array}$$ (38)

$$W^{\prime }\left(E\right)=W\left(E\right)/\int W\left(E\right)dE$$ (39)

The viscosity becomes

$$\begin{array}{c} \eta \left(0,T\right)\propto \langle t\rangle =b^{-1}\int W^{\prime }\left(E\right)\exp\left(+\left|E\right|kT\right)dE\\ =\int W^{\prime }\left(E\right)\tau \left(E\right)dE \end{array}$$ (40a)

The right-hand-side of Eq. (40a) is closely related to the partition function. We develop the consequences of this in Sec. 2.6.

In Secs. 2.4.2 and 2.4.3 we presumed that the process of flow could occur if only one particle jumped out of its well. But suppose it is required that within a space of a given volume there needs to be *M* particles that have simultaneously jumped out of their wells in order to have flow. It is shown in Appendix C that Eq. (40a) is generalized to

$$\begin{array}{c} \eta \left(0,T\right)\alpha {\langle t\rangle }^{M}={\left[b^{-1}\int W^{\prime }\left(E\right)\exp\left(+\left|E\right|/kT\right)dE\right]}^{M}\\ ={\left[\int W^{\prime }\left(E\right)\tau \left(E\right)dE\right]}^{M}. \end{array}$$ (40b)

This allows us to express the temperature dependence of *η* as

$$\log\eta \left(0,T\right)=B+M\log\left[\int W^{\prime }\left(E\right)\exp\left(+\left|E\right|/kT\right)dE\right]$$ (40c)

where *B* and *M* are considered to be constants.

#### 2.4.4 *D*(0, *T*) When the Wells Are of Different Depths

We now seek to calculate the diffusion coefficient when we have a distribution in well depths. The answer to this can be obtained by solving Eqs. (27) or (17), but we are unable to do this presently. Instead, we argue that the diagram of Fig. 2 which is our model for real glasses can be approximated under certain circumstances by the simpler diagram with all wells being of equal depth provided we choose an effective well depth. We choose for this effective well an effective rate constant *A*_eff_ given by

$$\left(\Sigma W_{i}\right)/A_{\mathrm{eff}}=\Sigma W_{i}/A_{i}.$$ (41)

The form of Eq. (41) reduces to the proper limiting form when there is only one well depth and additionally allows the escape from very deep wells to be the rate determining steps. The *W_i_* appear as shown because the number of times a particle falls into a well of depth *E_i_* is given by *W_i_*. The argument for this is that as soon as a configuration point escapes its well, because of the large value of *D* while running about in the upper wells it has exposed itself to the other wells, and because *b* is independent of *x* it falls into each well with equal probability. If the number of wells of depth *j* is *W_j_* the configuration point falls into a well of energy *E_j_* with a probability *W_j_* and then tries to escape with a probability proportional to *A_j_*. Thus, we know that *W_j_* is proportional to the number of well of type *j* and the effective diffusion coefficient for Fig. 2 is then given by

$$D_{\mathrm{eff}}=A_{\mathrm{eff}}D/b.$$ (42)

### 2.5 Evaluation of the Frequency Dependent ***η* (*ω, T*), *ε* (*ω, T*) and *D*(*ω, T*)**

#### 2.5.1 Evaluation of *η* (*ω, T*)

Equation (10b) has its analogue in polymer physics The complex viscosity is

$$\eta *\left(\omega ,T\right)=G\left(\omega ,T\right)=\int_{0}^{\infty }\exp\left(-i\omega t\right)g\left(t,T\right)dt$$ (43)

and the frequency dependent shear modulus is defined as

G*(ω,T)=iωη*(ω,T). (44)

At zero frequency we showed that

$$\eta *\left(0,T\right)\propto \langle t\rangle =\int_{0}^{\infty }\left(t\tau^{-1}\right)\exp\left(-t/\tau \right)dt=\tau .$$ (45)

But it would be wrong to identify *g*(*t*,*T*) with the integrand of Eq. (45). In fact since

$$\int_{0}^{\infty }\left(1/n!\right){\left(t\tau^{-1}\right)}^{n}\exp\left(-t/\tau \right)dt=\tau$$ (46)

any value of *n* would be permitted if the sole criterion were that the integral equal *τ*. Formulated in this way it is obvious that *n* = 0 gives the correct *g*(*t*,*T*) since it corresponds to a Maxwell element. Thus *g*(*t*,*T*) is proportional to $\int_{0}^{t}tP\left(t,T\right)dt$ and since the value of *g*(0,*T*) is *G*_0_ we have

$$g\left(t,T\right)=G_{0}\;\exp\left(-t/\tau \right).$$ (47)

This gives immediately

$$\eta *\left(\omega ,T\right)=G\left(\omega ,T\right)=G_{0}\tau /\left(1+i\omega \tau \right)$$ (48)

$$\eta *=\eta^{\prime }-i\eta^{\prime \prime },$$ (49)

$$\eta^{\prime }=G_{0}\tau /\left(1+\omega^{2}\tau^{2}\right)$$ (50a)

$$\eta^{\prime \prime }=G_{0}\omega \tau^{2}/\left(1+\omega^{2}\tau^{2}\right)$$ (50b)

while for a distribution *W′*(*E*) of well depths we obtain

$$\eta *\left(\omega ,T\right)=\int W^{\prime }\left(E\right)G_{0}\tau /\left(1+i\omega \tau \right)dE$$ (51)

$$g\left(t,T\right)=\int W^{\prime }\left(E\right)G_{0}\;\exp\left(-t/\tau \right)dE$$ (52)

$$\eta^{\prime }=\int W^{\prime }\left(E\right)G_{0}\;\tau \left(1+\omega^{2}\tau^{2}\right)dE$$ (53a)

$$\eta^{\prime \prime }=\int W^{\prime }\left(E\right)/\left(1+\omega^{2}\tau^{2}\right)dE.$$ (53b)

These relationships show clearly that non-Debye frequency behavior occurs because there is a distribution of relaxation times.

#### 2.5.2 Evaluation of Dielectric Response *ε* (*ωω T*)

Granted the calculation of the complex viscosity, the dielectric constant *ε* (*ω*< *T*) can also be obtained. Debye showed that if the dipoles are each imagined to be imbedded in the center of spheres (one dipole per sphere) that are in turn imbedded in a viscous fluid of viscosity *η* then the dielectric response is easily calculated [44]. Based on this result Di Marzio and Bishop showed [45] that if the viscous fluid has a complex viscosity *η* *(*ω*< *T*) then the formula is a simple generalization of the Debye formula, the only change being that *η* *(*ω*,*T*) replaces *η* (0, *T*). Thus,

$$\begin{array}{l} \left[\varepsilon \left(\omega ,T\right)-\varepsilon \left(\infty ,T\right)\right]/\left[\varepsilon \left(0,T\right)-\varepsilon \left(\infty ,T\right)\right]\\ ={\left(1+i\omega \eta *\left(\omega ,T\right)A\right)}^{-1} \end{array}$$ (54)

where *A* is a dimensional constant. The plus sign occurs in Eq. (54) because of our choice of the convention for the Fourier transform as in Eq. (43). This is consistent with Ferry’s [46] development of viscoelasticity for polymers.

#### 2.5.3 Evaluation of *D*(*ωω T*)

Equation (25a) shows that the diffusion coefficient *D* is a constant. In order for it to have a frequency dependence we would have to have had *∫D*(*t−τ)∂*^2^*N*/*∂x*^2^d*τ* for the first term on the right hand side of Eq. (25a). But, this is not the case. Equivalently we could have used the folding operation and written *D*(*t*−*τ*) = *Dδ*(*t*−*τ*). Further, from Eq. (29b) we see that the effective diffusion coefficient also has no frequency dependence at least to the quadratic approximation. Therefore, for our model we expect no frequency dependence in the diffusion coefficient.

$$D\left(\omega ,T\right)=b^{-1}\;\exp\left(-\beta \left|E\right|\right)D$$ (55)

For a distribution of well depths we have as before

D(ω,T)=D∫W(E)dE/[∫W(E)exp(+β|E|)dE]. (56)

### 2.6 Evaluation of *W*(*E*)

The above relationships are quite remarkable for they state that long time relaxations-viscosity, diffusion and dielectric response depend only on the well depths and the distribution of well depths. The only thing remaining is for us to evaluate *W*(*E*). Notice that if this can be done then our kinetics of glasses will depend only on the equilibrium statistical mechanics. For glasses statistical mechanics plus the principle of detailed balance is everything provided we are looking only at the long time behavior.

The classical and quantum mechanical partition functions are given by (we ignore the thermal wavelength)

$$Q_{\text{classical}}=\int \exp\left(-\beta E\left(..q_{j}..\right)\right)\pi dq_{j}$$ (57a)

$$Q_{Q.M}=\int \exp\left(-\beta E_{j}\right)d_{j}$$ (57b)

where the integral signs represent discrete sums and/or continuum integrals. By grouping together all states with the same energy we obtain

$$Q_{\text{classical}}=\int \exp\left(-\beta E\right)W\left(E\right)dE$$ (58a)

$$Q_{Q.M}=\int \exp\left(-\beta E\right)W\left(E\right)dE$$ (58b)

which are identical in form to the argument of the logarithm on the RHS of Eq. (40c). Using the formula *F*_c_ = −*kT*ln*Q* which connects the configurational part of the Helmholtz free energy *F*_c_ to the partition function *Q* we have immediately

$$\log\eta \left(0,T\right)=B-M^{\prime }F_{c}/kT.$$ (59)

This remarkable formula which relates viscosity to free energy is very different from the Vogel-Fulcher-Tammann-Hesse form [47], the Bendler-Shlesinger form [48], the Avramov form [49], the Adam-Gibbs form [12] or the mode coupling theory result [50]. We discuss it in Sec. 3.1.

The frequency dependent viscosity, given by Eqs. (53), cannot be expressed as a function of free energy. Rather, we first must determine *W*(*E*) separately before we can evaluate *η* (*ω*< *T*). If in Eq. (58a) we choose the lowest energy as our zero of energy, then exp(−*βF*(*β*)) is the Laplace transform of *W*(*E*) and *W*(*E*) is the inverse transform of exp(−*βF*(*β*)).

Another approach is to use the results of Stillinger who suggests that *W*(*E*) is given by [51, 52]

$$W\left(E\right)=\exp\left(-\theta \;{\left(E-E_{0}\right)}^{2}\right).$$ (60)

With this substitution the time dependent shear modulus, Eq. (52), reads

$$\begin{array}{c} g\left(t,T\right)=\int \exp\left(-\theta \;{\left(E-E_{0}\right)}^{2}\right)\\ \exp(-bt\;\exp\left(-\beta \left|E\right|\right)dE/\;\int \exp\left(-\theta \;{\left(E-E_{0}\right)}^{2}\right)dE. \end{array}$$ (61)

The time dependent behavior of Eq. (61) is closely related to that of the “after-effect function” tabulated by Janke and Emde [53]. As shown previously the after-effect function has a time dependence which looks very much like the stretched exponential function [23]. In fact, Stillinger, starting from the empirically observed stretched exponential form for relaxation shows that the Gaussian form for *W*(*E*) is implied [52].

## 3. Discussion and Conclusions

### 3.1 Discussion of Results

Equation (59) which connects viscosity to free energy is remarkable in several respects. First, it states that the viscosity *and its temperature derivative* are continuous as we proceed through the transition. We had, in Sec. 1.8, used the argument that the dissipative quantities should have the same transition behavior as the thermodynamic variables. So, for a first-order transition the viscosity is discontinuous through the transition because the entropy and volume are. But we have now obtained the result that for a second-order transition the viscosity does not show a break as we traverse the transition point. In the past various groups have argued that the volume [54] is the controlling quantity, or the enthalpy [55], or the entropy [1–5]. We are claiming that the entropy theory of glass formation, which is merely a theory that locates the transition in temperature and pressure space as a function of the molecular parameters such as chain length, intermolecular energies and intramolecular stiffness energies etc. (see Sec. 1.4) can be extended to include slow motion kinetics. When this is done the *only* determinate of the kinetic aspects of glass formation in the limit of zero frequency is the thermodynamic free energy! See Eq. 62e. However, as Eqs. (51–54) show this is not true for the frequency dependent dissipative quantities.

The Vogel-Fulcher-Tammann-Hesse form [47] from which the WLF equation [56] is easily derived is

$$\log\eta =B+A/\left(T-T_{0}\right).$$ (62a)

The Bendler-Shlesinger form [48] is

$$\log\eta =B+A/{\left(T-T_{0}\right)}^{1.5}$$ (62b)

The Avramov form [49] is

$$\log\eta =B+0.434{\left(A/T\right)}^{\alpha }$$ (62c)

and the Adam-Gibbs form [12] is

$$\log\eta =B+A/TS_{c}.$$ (62d)

These forms should be compared to our form which is

$$\log\eta =B-AF_{c}/kT.$$ (62e)

We will not discuss the mode coupling form for viscosity since we accept the argument [57] that the implied singularity is considerably higher than *T*_g_.

Although each of the first four forms has some theoretical underpinning it is probably true that the reason they fit experimental data well is that they (the first three) are three-parameter fits and the viscosity curves are rather structureless to begin with. To see that it is not terribly significant to fit a curve of relatively little structure with three parameters imagine *B* to locate the curve vertically, another of the parameters stretches the curve so that there is a fit at both high and low temperatures. Finally the third parameter gives the curve the proper amount of curvature. Viewed in this way we see that the fact that formulas of different construction give decent fits to the data is not surprising. A real test of the theories is whether they can determine the values of the three parameters from theory.

Viewed from this perspective the last two Eqs. (62d), and (62e) are more significant because they contain one less parameter. The original GD lattice theory can be used to obtain *F*_c_. A real theory should contain no parameters. Schroedinger’s equation plus the laws of statistical mechanics should be sufficient. The authors intend to examine the meaning of the *B* and *A* parameters of Eq. (62e) in a subsequent paper. For now we will merely comment on the implication of the form of our equation, assuming *A* and *B* to be temperature independent.

Angell’s classification [7] of glasses into strong and fragile receives an easy interpretation from Eq. (62e). First, we need to use the experimental value of the free energy in Eq. (62e). There is a general consensus that the specific heat break at the glass transition, *C*_p,c_, varies inversely with temperature [58]. We therefore use the form *C*_p,c_ = *α*/*T*.

$$C_{p,c}=\alpha /T\to S_{c}=\alpha \left(1/T_{2}-1/T\right)\to$$ (63)

$$F_{c}=-C-\alpha \left(T/T_{2}-1\right)+\alpha \ln\left(T/T_{2}\right),T_{2}\leq T$$ (64a)

$$F_{c}=-C,T\leq T_{2}$$ (64b)

where the constant of integration *C* is (part of) the energy of activation.

To obtain these equations we integrated *C*_p,c_ = *T∂S*_c_/*∂T, S*_c_ = *∂F*_c_/*∂T* and ignored any pressure dependence. Below The transition temperature *T*_2_ the configurational entropy is zero according to the simple version of the GD theory so that we have only energy of activation while above *T*_2_ the specific heat is assumed to decrease inversely with temperature in accord with experiment.

Using Eq. (62e) we can eliminate *B* by choosing a reference temperature T* for which the viscosity equals 10^13^ poise. A little algebra results in

$$\begin{array}{l} \log\eta =13+\zeta x\ln x+\left(1-x\right)\\ \times \left(\zeta \left[1+\ln\left(T*/T_{2}\right)\right]-\theta \right) \end{array}$$ (65a)

$$\partial \log\eta /\partial x=\theta -\zeta \ln\left(T*/T_{2}\right)+\zeta \ln x$$ (65b)

$$\begin{array}{c} \partial^{2}\log\eta /\partial x^{2}=\zeta /x,T_{2}\leq T*\leq T,\\ T_{2}\leq T\leq T* \end{array}$$ (65c)

$$\begin{array}{c} \log\eta =13+\theta \left(x-1\right)-\zeta \left[T*/T_{2}-1\right]\\ +\ln\left(T*/T_{2}\right) \end{array}$$ (66a)

logη/∂x=θ (66b)

$$\partial^{2}\log\eta /\partial x^{\prime }2=0,\;\;T\leq T_{2}\leq T*$$ (66c)

$$\log\eta =13+\theta \left(x-1\right)+\zeta \left[\left(T*/T_{2}-x\right)+x\ln\left(xT_{2}/T*\right)\right]$$ (67a)

$$\partial \log\eta /\partial x=\theta -\zeta \ln\left(T*/T_{2}\right)+\zeta \ln x$$ (67b)

$$\partial^{2}\log\eta /\partial x^{2}=\zeta /x,\;\;T*\leq T_{2}\leq T$$ (67c)

logη=13+θ(x−1) (68a)

∂logη/∂x=θ (68b)

$$\begin{array}{c} \partial^{2}\log\eta /\partial x^{2}=0,T\leq T*\leq T_{2},\\ T*\leq T\leq T_{2} \end{array}$$ (68c)

where *θ* = *CA*/kT*, *ζ* = *αA*/kT*, *x* = T*/*T*. T* is the temperature for which *η* = 10^13^ poise. If we had picked 10*^y^* as the reference viscosity then the above equations would be the same with *y* replacing 13 and T* being the temperature at which the viscosity is 10*^y^* poise.

Equations (65c,) and (67c) show that the curvature is positive (curve is concave up) and that the curvature is greater the larger the specific heat. Also, as the value of T*/*T* decreases the curvature is larger. Below the glass temperature we predict pure Ahrennius behavior. These features are also features of Angell’s classification of glasses into strong and fragile varieties. An interesting prediction is that if T*/*T*_2_ = 1 then the initial slope at T*/*T* = 1 is independent of specific heat. It does however depend on *C*.

**Fig. 7:**
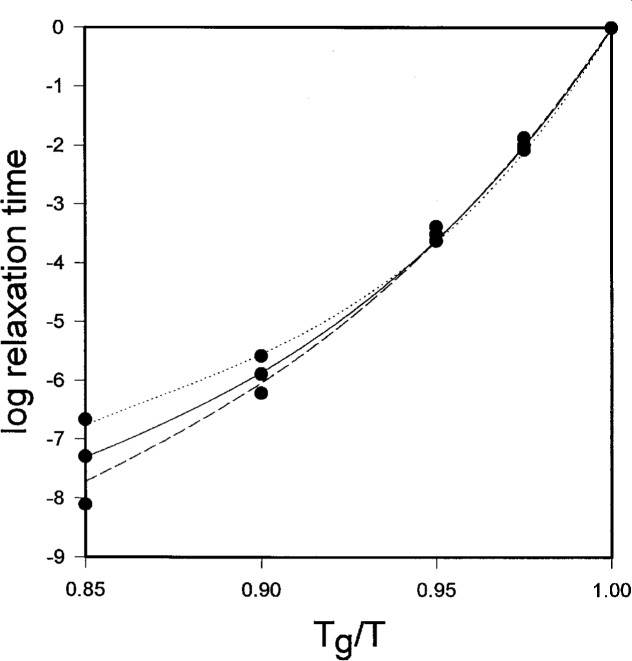
An attempt to explain the fragility plots of Angell. Using for the configurational free energy a form derived by assuming that the specific heat is proportional to *T*^−1^, which is in accord with experiment, we obtain a fit to the plots of log relaxation time versus *T*_g_/*T*. The curves are experimental data for polydimethylsiloxane of varying molecular weight, and the circles are predicted values. That 1) the curves all start with the same slope at T*/*T* = 1; 2) the curvature increases with decreasing T*/*T*; 3) the curvature increases with increasing specific heat are all predicted by our equation. See text.

We can test these predictions for polymers using data for polydimethylsiloxane of varying molecular weight. Roland and Ngai [59] using dielectric relaxation data of Kirst et al. [60] and specific heat data of Bershstein and Egorov [61] created fragility plots of the logarithm of relaxation time versus *T*_g_/*T* where *T*_g_ was defined as the temperature for which the relaxation time was one second. These curves which are reproduced in Fig. 7 show, as Roland and Ngai observed, 1) that the slope of the curves at T*/*T* = 1 are independent of specific heat—we predict this, 2) The curvature is larger the smaller the value of *T*_g_/*T*—we predict this, and 3) the curves flare out for low *T*_g_/*T* with the higher specific heat (low molecular weight) material flaring up and the low specific heat (high molecular weight) material flaring down—we predict this. The filled circles are our numerical predictions. We chose *A* and *B* to fit the center curve. We then scaled *ζ* by the ratio of the specific heats for the low and high molecular weight polymers to obtain the upper and lower points at each temperature. Our fits assume that *C* is independent of molecular weight.

We also give the formulas for the case that the configurational specific heat is constant above *T*_2_. Our reason for doing this is that although the GD lattice model predicts that the configurational specific heat approaches zero as the temperature increases it does not do so with purely inverse temperature dependence. So, a combination of the two specific heat variations may better fit the experimental data.

$$C_{p,c}=\alpha^{\prime }$$ (69)

$$S_{c}=\alpha^{\prime }\ln\left(T/T_{2}\right),\;\;\;T_{2}\leq T$$ (70a)

$$S_{c}=0,\;\;\;T\leq T_{2}$$ (70b)

$$F_{c}=-\alpha^{\prime }T\ln\left(T/T_{2}\right)+\alpha^{\prime }\left(T-T_{2}\right)-C,\;\;T_{2}\leq T$$ (70a)

$$F_{c}=-C,\;\;\;T\leq T_{2}$$ (70b)

If we again define T* as the temperature for which the viscosity equals 10^13^ poise we obtain

$$\log\eta =13-\zeta^{\prime }\ln\left(x\right)+\left(\zeta^{\prime }T_{2}/T*+\theta \right)\left(x-1\right)$$ (71a)

$$\partial \log\eta /\partial x=\zeta^{\prime }T_{2}/T*+\theta -\zeta^{\prime }x^{-1},$$ (72b)

$$\begin{array}{c} \partial^{2}\log\eta /\partial x^{2}=+\zeta^{\prime }x^{-2},\\ T_{2}\leq T*\leq T,\;\;T_{2}\leq T\leq T* \end{array}$$ (72c)

$$\begin{array}{c} \log\eta =13-\zeta^{\prime }\ln\left(T*/T_{2}\right)+\theta \left(x-1\right)\\ +\zeta^{\prime }\left(1-T_{2}/T*\right), \end{array}$$ (73a)

∂logη/∂x=θ, (73b)

$$\partial^{2}\log\eta /\partial x^{2}=0,\;\;T\leq T_{2}\leq T*$$ (73c)

$$\begin{array}{c} \log\eta =13+\zeta^{\prime }\ln\left(T/T_{2}\right)+\theta \left(x-1\right)\\ +\zeta^{\prime }\left(T_{2}/T*\right)\left(x-T*/T_{2}\right) \end{array}$$ (74a)

$$\partial \log\eta /\partial x=\zeta^{\prime }T_{/}T*+\theta -\alpha^{\prime }x^{-1},$$ (74b)

$$\partial^{2}log\eta /\partial x^{2}=+\zeta^{\prime }x^{2},\;T*\leq T_{2}\leq T$$ (74c)

logη=13+θ(x−1), (75a)

logη/∂x=θ, (75b)

$$\partial^{2}\log\eta /\partial x^{2}=0,\;\;T*\leq T\leq T_{2},\;T\leq T*\leq T_{2}$$ (75c)

where *ζ* = *α*′*A*/*k* and *θ* = *CA*/kT*. These curves again show the features of the strong-fragility plots discussed by Angell.

It should be noted that if either of the above forms for the entropy is substituted into the Adam-Gibbs form [Eq. (62d)] one obtains a decreasing slope with increasing specific heat at T*/*T* = 1. Also the curvature of the log*η* vs T*/*T* curve becomes smaller as T*/*T* decreases which is contrary to the sense of virtually all experimental results.

Can T* ever be less than *T*_2_? Under the paradigm of the Vogel-Fulcher equation this is a foolish question. However, since the viscosity and its derivative are, according to Eq. (62a), continuous through the second-order transition and since the viscosity is never infinite *T*_2_ can not be located accurately by measurements of viscosity; we see no reason why it can not be greater than T*. The possibility that *T*_2_ corresponds to a finite viscosity may well be masked by the process of falling out of equilibrium which can be discussed only by examining the time or frequency dependent viscosity.

The new formulas for viscosity suggests several new directions. First, an examination of the way which *C*/T* varies with material should be made.

We remark that these questions are equilibrium thermodynamic and statistical mechanical questions so that their investigation should not be difficult. For systems with constant *C* the initial slope of the curve at T*/*T* = 1 would be inverse to T*. Also systems for which the motion is highly cooperative would show a higher *C*. Systems which have the same scaled potential energy surface, i.e., *hE*(.. *q_i_*..) where *h* is any constant, should display superposed fragility plots. Such systems which have no specific heat break at *T*_g_ should all superpose with the form of a straight line. Finally how the specific heat α relates to *C*/T* should be examined.

Another possibility that deserves serious consideration is that the parameters *B* and *A* have a temperature dependence which must be added to that of the free energy. This thought is consistent with the view expressed by some that the temperature dependence of viscosity and diffusion at higher temperatures is adequately addressed by mode coupling theory and that the behavior over the full temperature range can be obtained by a cross-over treatment that combines the high temperature mode coupling theory with a theory of low temperatures such as has been presented here.

We leave such a development to the future.

### 3.2 Conclusions

This paragraph describes the logic of our development. We first observed that there must exist at low temperatures an equilibrium glass phase because the crystal phase is not ubiquitous. It is only for systems that can crystallize that the glass phase can be considered to be a metastable phase. We next showed that the Gibbs-Di Marzio (GD) theory [1–5] which postulates that the glass transition occurs when the configurational entropy approaches zero locates the glass transition correctly in temperature-pressure space for a wide variety of experiments. It also resolves the Kauzmann paradox [6]. We next observed that the correct equilibrium theory of those materials that form glasses, whatever it may be, must be used as a groundform onto which a proper kinetic theory of glasses is constructed. The connection between kinetics and equilibrium was then made via the principle of detailed balance which relates the ratio of the rates for jumping to and from a pair of states to the free energy difference between the states. This law when combined with the observation that the configuration point of a glass system spends most of its time in deep potential energy minima allows us to construct a minimal model (a trapping model) which can be solved in some limiting cases. We thereby obtained formulas for the complex viscosity *η* *(*ω, T*) and shear relaxation modulus g(*t, T*), the diffusion coefficient *D*(*ω, T*) = *D*(0, *T*) and the dielectric response *ε* (*ω, T*).

Our relaxation modulus has the form of the after-effect function tabulated by Jahnke and Emde [53] which we had obtained previously [32]. Its behavior is very close to the stretched exponential form.

More surprising is our formula relating the zero frequency viscosity *η* to the configurational part of the thermodynamic Helmholtz [62] free energy *F*_c_

$$\log\eta =B-AF_{c}/kT$$

It is surprising that the viscosity is continuous through the transition. If this conclusion holds, viscosity or other dynamical measurements may be the worst way to locate glass temperatures. The use of thermodynamic quantities which show breaks in slope should be preferred. Initially we had expected (naively in retrospect) that there should be a break in slope of *η* (*T*) vs *T*. Below the transition the behavior is Arrhenius corresponding to the fact that there is energy but not entropy of activation below the transition. Above the glass transition entropy of activation kicks in.

When sensible approximations for *F*_c_ are used this formula displays the main features of the strong-fragile glass classification scheme proposed by Angell [7]. Glasses with small specific heat breaks at the glass transition show little curvature on log*η* versus inverse temperature plots while glasses with large specific heat breaks show positive curvature. See Sec. 3.1.

We have not yet examined the temperature dependence of *B* and *A* in the above equation. An approach to this problem is to excise from phase space those phase points corresponding to deep potential energy minima and solve the kinetics of such a circumscribed space. Since the resulting equations should be applicable to the high temperature side of the glass transition it may be that mode-coupling theory can be used for this part of the problem. A theory of glasses that is valid over a wide range of temperatures undoubtedly requires incorporation of vibrational properties.

## 4. Appendix A: A Particle Falling Back into a Well From Which It Is Trying to Escape Retains Its Exponential Distribution

Let a particle have a normalized probability distribution *p*(*t*) for escaping from a well. But after it has left the well let there be a probability 1 − *ϕ* that it fall back into the well and *ϕ* that it escape permanently on that attempt. Then the particle can escape permanently after it left for the first time, or the second time or the third time, etc. The true distribution function for escape is

$$\begin{array}{l} p_{\text{true}}=p\left(t\right)\phi +\int \underset{t_{1}+t_{2}=t}{p\left(t1\right)(1}-\phi )p\left(t_{2}\right)dt_{1}\;dt_{2}\phi \\ \;+\int \underset{t_{1}+t_{2}+t_{3}=t}{p\left(t_{1}\right)p\left(t_{2}\right)(1}-\phi {)}^{2}p\left(t_{3}\right)dt_{1}\;dt_{2}\;dt_{3}\phi +\ldots \end{array}$$ (A1)

The integrals are recognized to be folding operations. Denoting the Laplace transform of *p* by *P* and taking the Laplace transform of Eq. (A1) we obtain

$$\begin{array}{c} P_{\text{true}}=P\phi +P^{2}\left(1-\phi \right)\phi +P^{3}{\left(1-\phi \right)}^{2}\phi \\ +P^{4}{\left(1-\phi \right)}^{3}\phi +\ldots \\ =P\phi /\left(1-\left(1-\phi \right)P\right) \end{array}$$ (A2)

This equation states that if *p* is normalized, so that *P*(0) = *∫ p*(*t*)d*t* = 1), then *p*_true_ is also normalized.

For the special case where *p* is exponential in *t* we have

$$p\left(t\right)=\tau^{-1}\;\exp\left(-t/\tau \right),\;\;P=P\left(s\right)=\tau^{-1}/s+\tau^{-1}).$$ (A3)

Using Eq. (3) in Eq. (2) we obtain

$$P_{\text{true}}=P_{\text{true}}\left(s\right)=\phi \tau^{-1}/\left(s+\phi \tau^{-1}\right)$$ (A4)

which on transforming back to the time domain gives

$$P_{\text{true}}=\phi \tau^{-1}\;\exp\left(-\phi t/\tau \right)$$ (A5)

Thus, the final distribution remains exponential and is normalized but the time constant for exiting the well is increased by the factor *ϕ*^−1^.

## 5. Appendix B. Calculation of the Shear Viscosity *η* (0,*T*)

To calculate the shear viscosity consider a material between two parallel plates one of which is fixed and one of which moves under a shear force

σ/〈v〉=η(0,T) (B1)

But

$$\langle v\rangle =x/t_{f}=V\left(t_{f}-t_{i}\right)/t_{f}=V\Delta t/t_{f}$$ (B2)

where *V* is the velocity that the plate has for the time interval *t*_f_ − *t* i = Δ*t* during which the system is flowing in the configurational sea. From time zero to *t*_i_ there was no motion because the particle was in one of the deep wells. We obtain

$$\begin{array}{l} \eta \left(0,T\right)=\left(\sigma \left(0,T\right)/V\right)t_{f}/\Delta t=\eta_{c}t_{f}/\Delta t\\ =\eta_{c}\left(t_{i}+\Delta t\right)/\Delta t=\eta_{c}\left(1+t_{i}/\Delta t\right). \end{array}$$ (B3)

This process of sticking and slipping is imageined to happen over and over again. The distribution function *P*(*t*_i_, *T*) for jumping out of a well at time *t*_i_ when all the deep wells have the same depth is given by

$$P\left(t_{i},T\right)=\tau^{-1}\;\exp\left(-t/\tau \right),=b^{-1}\;\exp\left(+\beta \left|E\right|\right).$$ (B4)

Thus over a period of time sufficiently large to allow for many visits to the deep wells we have

$$\begin{array}{c} \eta \left(0,T\right)=\eta_{c}\left(1+\langle t\rangle /\Delta t\right)\approx \left(\eta_{c}/\Delta t\right)\langle t\rangle \\ =\left(\eta_{c}/\Delta t\right)\tau \end{array}$$ (B5)

which proves our contention that the viscosity is proportional to the average residence time spent in deep wells.

Evidently, if there is a distribution of well depths, *W*′(*E*), normalized so that the *∫ W*′(*E*)d*E* = 1, we obtain

$$\begin{array}{c} \eta \left(0,T\right)\approx \left(\eta_{c}/\Delta t\right)\int \int W^{\prime }\left(E\right)tP\left(t,T\right)dEdt=\\ \left(\eta_{c}/\Delta t\right)\int \int W\left(E\right)b^{-1}\;\exp\left(+\beta \left|E\right|\right)dE/\int W\left(E\right)dE. \end{array}$$ (B6)

Given the exponential character of *P*(*t, E*) we have succeeded in relating the zero frequency viscosity *η* (0, *T*) to the two integrals which are purely equilibrium quantities.

## 6. Appendix C. What if Flow Requires Several Particles to Be Out of Their Wells Simultaneously

It may be unreasonable to suppose that flow can occur in the region between two parallel plates when one particle only is out of its well. Certainly, as the amount of the material between the plates is increased it is more reasonable to expect that flow requires that the number of particles simulaneously out of their wells be proportional to the volume of material. We propose that the number required per unit volume be some large number *M*.

Let us begin by considering the case where there is flow only if two particles have simultaneously escaped the wells. Consider one particle jumping out of a well at time *t*_i_, cruising the configurational sea for a time interval Δ*t* = *t*_f_−*t*_i_, falling into a well, and then starting the process all over again. One can imagine these time intervals placed stochastically on the positive infinite half line. Obviously flow for the system will occur only when there is an overlapping of the Δ*t*’s of one particle with the Δ*t*’s of the other. The fraction of time that these time intervals overlap is obviously given by (*n*Δ*t*/Σ(*t*_i_ + Δ*t*))^2^. We imagine the particles to have jumped out of the wells *n* times, where *n* is very large. Refering to appendix B we calcuate the average velocity to be 〈*v*〉 to be

$$\langle v\rangle =V{\left(n\Delta t/(\Sigma \left(t_{i}+\Delta t\right)\right)}^{2}.$$ (C1)

The viscosity is

$$\begin{array}{c} \eta =\sigma /\langle v\rangle =\left(\sigma /V\right)\cdot {\left[\left(\Sigma t_{i}+n\Delta t\right)/n\Delta t\right]}^{2}\\ =\eta_{c}{\left[1+\Sigma t_{i}/n\Delta t\right]}^{2}. \end{array}$$ (C2)

Now if we require *M* particles to be simultaneously out of the wells in order to have flow we need only realize that the probability for this is

$${\left[n\Delta t/\Sigma \left(t_{i}+\Delta t\right)\right]}^{M}.$$ (C3)

Thus

$$\eta =\eta_{c}{\left[1+\Sigma t_{i}/n\Delta t\right]}^{M}.$$ (C3)

Now if *M* or more particles are needed to be out of the wells simultaneously we have for the probability (fraction of time) for this to occur

$$\begin{array}{c} {\left[1+\Sigma t_{i}/n\Delta t\right]}^{M}+{\left[1+\Sigma t_{i}/n\Delta t\right]}^{M+1}\\ +{\left[1+\Sigma t_{i}/n\Delta t\right]}^{M+2}+\ldots \end{array}$$ (C4)

which leads to

$$\begin{array}{c} \eta =\eta_{c}{\left[1+\Sigma t_{i}/n\Delta t\right]}^{M}\left[1+n\Delta t/\Sigma t_{i}\right]\\ \approx \eta_{c}{\left[1+\Sigma t_{i}/n\Delta t\right]}^{M}. \end{array}$$ (C4)

We now need to evaluate

$$\Sigma t_{i}=n\int t\tau^{-1}\;\exp\left(-t/\tau \right)dt=n\tau .$$ (C5)

But if there is a distribution of well depths then

$$\begin{array}{c} \Sigma t_{i}=n\frac{\int W\left(E\right)t\tau^{-1}\;\exp\left(-t/\tau \right)dtdE}{\int W\left(E\right)dE}\\ =n\frac{\int W\left(E\right)\tau \left(E\right)dE}{\int W\left(E\right)dE}=n\langle \tau \rangle \\ =n\frac{\int W\left(E\right)b^{-1}\;\exp\left(+\beta \left|E\right|\right)dE}{\int W\left(E\right)dE}. \end{array}$$ (C6)

Therefore

$$\begin{array}{c} \eta =\frac{\eta_{c}{\left[1+\int W\left(E\right)b^{-1}\;\exp\left(+\beta \left|E\right|\right)dE\right]}^{M}}{\int W\left(E\right)dE\Delta t}\\ \approx \frac{\eta_{c}{\left(\int W\left(E\right)b^{-1}\;\exp\left(+\beta \left|E\right|\right)dE\right)}^{M}}{\int W\left(E\right)dE\Delta t}. \end{array}$$ (C7)

By taking the logarithm of this equation we can cast it into a form usually used to compare with experiments and those equations created to explain experiments such as The Vogel-Fulcher law [47], the Bendler-Shlesinger law [48], and the Avramov law [49].

logη=B+Mlog(∫W(E)exp(+β|E|)dE) (C8)

$$B=\log\eta_{c}-M\log\left(b\int W\left(E\right)dE\Delta t\right).$$ (C9)

In this paper we treat *B* as a constant in order to focus on the temperature dependence of the second term on the RHS of Eq. (C8). Discussion of the temperature dependence of *B* is reserved for future work.
